# Biological and mechanical influence of three-dimensional microenvironment formed in microwell on multicellular spheroids composed of heterogeneous hair follicle stem cells

**DOI:** 10.1038/s41598-023-49510-6

**Published:** 2023-12-20

**Authors:** Seungjin Lee, Nackhyoung Kim, Sung-Hwan Kim, Soo-Jong Um, Joong Yull Park

**Affiliations:** 1 Department of Mechanical Engineering, Graduate School, Chung-Ang University, 84 Heukseok-ro, Dongjak-gu, Seoul, 06974 Republic of Korea; 2 Department of Integrative Bioscience and Biotechnology, Sejong University, 209 Neungdong-ro, Gwangjin-gu, Seoul, 05006 Republic of Korea; 3 Cellsmith Inc., 38 Pungseong-ro, Gangdong-gu, Seoul, 05393 Republic of Korea; 4 Department of Intelligent Energy and Industry, Graduate School, Chung-Ang University, Seoul, 06974 Republic of Korea

**Keywords:** Biotechnology, Cell biology, Stem cells, Health care, Engineering

## Abstract

Hair loss caused by malfunction of the hair follicle stem cells (HFSCs) and physical damage to the skin is difficult to recover from naturally. To overcome these obstacles to hair follicle (HF) regeneration, it is essential to understand the three-dimensional (3D) microenvironment and interactions of various cells within the HFs. Therefore, 3D cell culture technology has been used in HF regeneration research; specifically, multicellular spheroids have been generally adapted to mimic the 3D volumetric structure of the HF. In this study, we culture HF-derived cells, which are mainly composed of HFSCs, in the form of 3D spheroids using a microwell array and discuss the effects of the 3D cellular environment on HF morphogenesis by expression measurements of Sonic hedgehog signaling and stem cell markers in the HF spheroids. Additionally, the influences of microwell depth on HF spheroid formation and biological conditions were investigated. The biomolecular diffusion and convective flow in the microwell were predicted using computational fluid dynamics, which allows analysis of the physical stimulations occurring on the spheroid at the micro-scale. Although a simple experimental method using the microwell array was adopted in this study, the results provide fundamental insights into the physiological phenomena of HFs in the 3D microenvironment, and the numerical analysis is expected to shed light on the investigation of the geometric parameters of the microwell system.

## Introduction

Hairs are produced in complex mini-organs called hair follicles (HFs). Malfunctions of the HF stem cells (HFSCs) and physical damage to the skin cause hair loss, which is difficult to recover from naturally. Accordingly, various in vitro cell culture techniques have been used to restore the damaged HF functions and analyze the effects of various biochemical stimuli (hormones, growth factors related to folliculogenesis, culture substrates, heterogenic cell–cell and cell–matrix interactions, etc.) on HF cells (HFCs)^1^. Recently, the physiological characteristics of HFs have been studied using three-dimensional (3D) cell culture methods^2^. Here, the cellular functions are expressed by the effects of the in vivo biochemical stimuli in the 3D microenvironment. Therefore, replicating the 3D tissue environment has been proposed as an effective method for investigating HF cell physiology.

The HF cycle is a result of the complex interactions among various types of cells in the 3D microenvironment of the HF, and the mechanisms that constitute this phenomenon are still unclear^3^. The multicellular spheroid culture, which is a technique that effectively mimics the 3D cellular microenvironment, has contributed to HF research. In particular, investigation of the functions of the dermal papilla cells (DPCs) is a main issue in regulating the HF cycle. This methodology has been expanded to studies on various cell types involved in HF regeneration, such as human adipose-derived stem cells (hASCs)^4^, HFSC^5^, and keratinocytes^2^. Nonetheless, to fully understand the representative in vivo environment of the HFs within the spheroids, it is essential to enhance the efficiency of the spheroid culture method and conduct further research on this technique.

In spheroid research, the microwell array system has been evaluated as an efficient spheroid culture method with the advantages such as ease of use, uniformity of spheroid formation, and mass production^6^ compared with other technologies, including the hanging drop^7^, low adherence substrate^8^, microfluidic encapsulation^9^, rotating bioreactor^10^, and magnetic manipulation^11^. As the microwell platform was developed further, modifications to the microwell were attempted to improve functionality, such as enhanced spheroid uniformity^6^, reduced cell loss^12^, and controlled interactions between the external environment and spheroids^13–15^. Although the design functionalities of the microwell have been successfully demonstrated in previous studies, they have consequently led to derivation of several geometrical parameters from these functional structures. Moreover, there is insufficient discussion on the biological and mechanical effects of several design parameters proposed in each research, and the applications of these systems have been mostly limited to laboratory-scale experiments. To overcome these limitations, detailed parametric analyses are essential.

**Figure 1:**
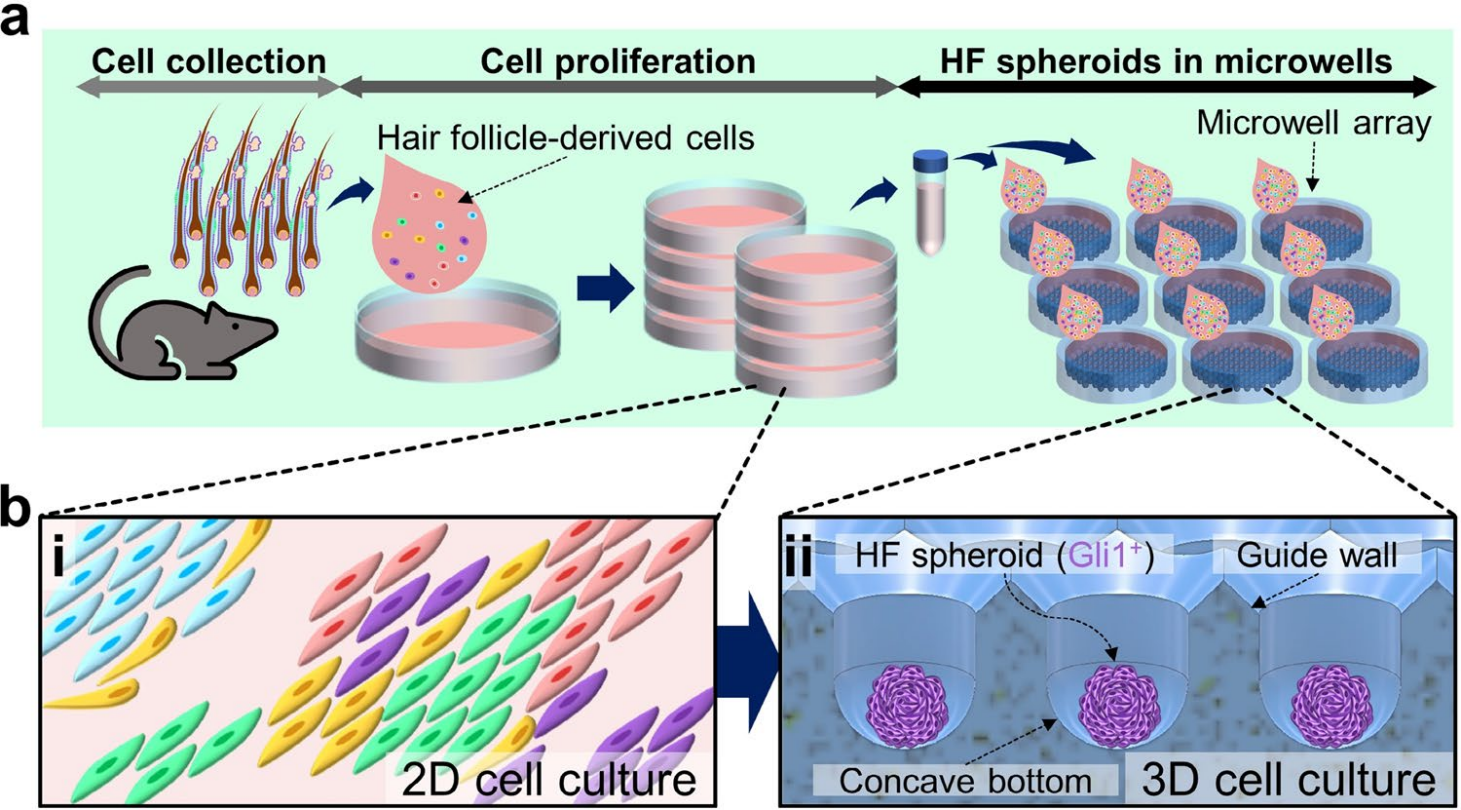
Cultivation of multicellular spheroids composed of hair-follicle-derived cells. (**a**) Process of HF spheroid cultivation using the microwell array. (**b**) Comparison of heterogeneous HF cell characteristics between (i) 2D culture conditions in a culture plate and (ii) 3D spheroid microenvironment in the microwell array.

In the present study, HF spheroid generation using overall cells derived from the HFs and their potential are discussed. All types of HF-derived cells were collected and grown in culture plates to obtain sufficient amounts for experimentation, and the microwell array was used for efficient formation of HF spheroids (Fig. 1a). The heterogeneous HFCs form spheroids in the microwell array, and their characteristics are modified by the influence of the 3D microenvironment (Fig. 1b). We investigated the influence of the microwell depth, which is one of the fundamental shape parameters, as well as the phenomenon of heterotypic HFCs occurring in the 3D microenvironment of the spheroid. The observations of the HF spheroids were conducted in early stages of cell cultivation, and the cell markers related to the HF cycle were measured. Additionally, computational fluid dynamics (CFD) analysis was performed to further focus on the fluid dynamic effects of the microwell depth. The findings of this study on the cultivation of heterogeneous HF spheroids is expected to serve as a scientific basis for improving the efficiency of HF research and for advancing the understanding of HF regeneration.

## Materials and methods

### Fabrication of microwell array

**Figure 2:**
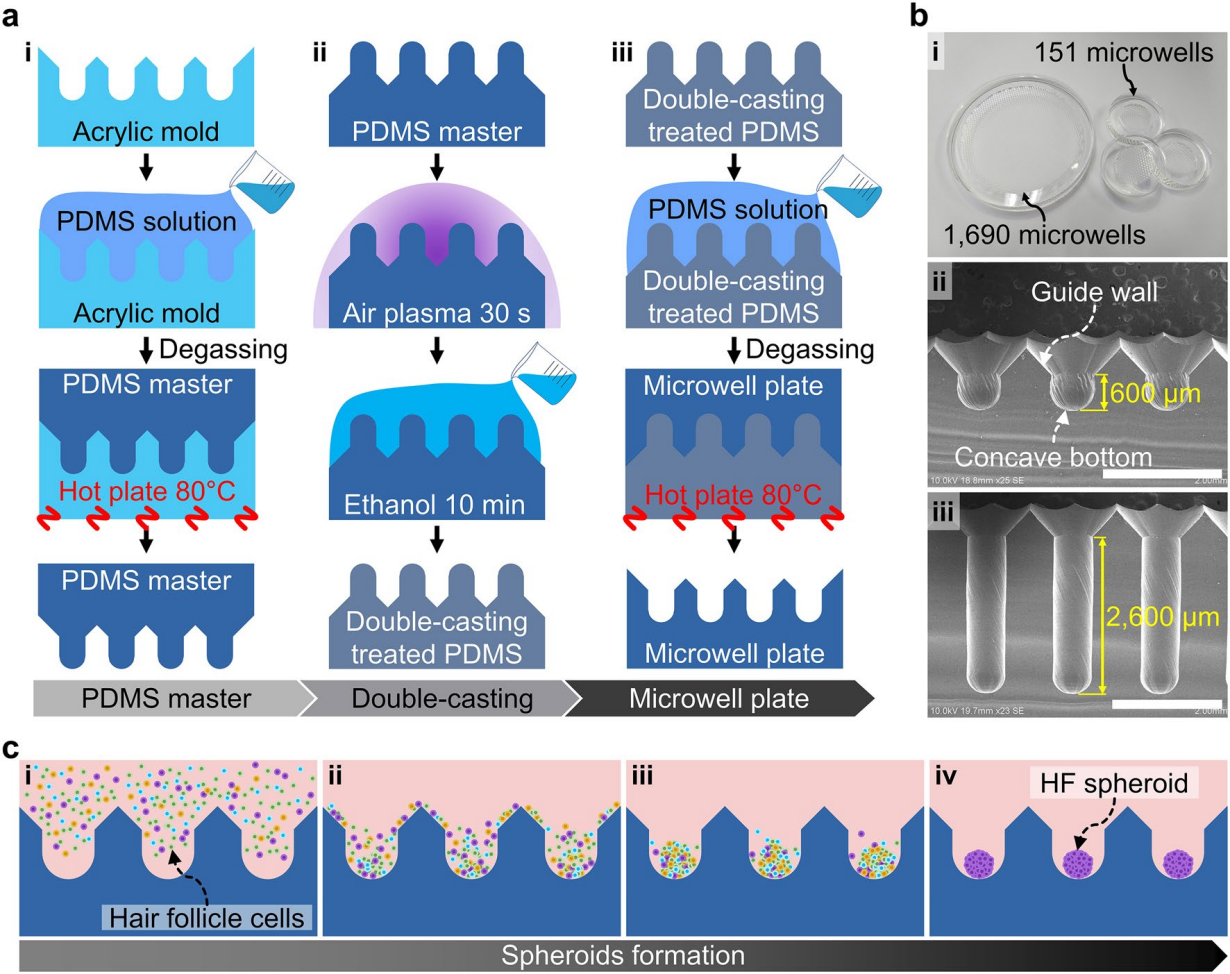
Fabrication of the microwell plate and spheroid formation. (**a**) Casting method of the PDMS microwell plate. (i) The PDMS master was cast using an acrylic microwell mold. (ii) The PDMS master was then treated by the double-casting process consisting of ethanol and air plasma treatments. (iii) Using the double-casting treated PDMS master, the microwell plate was cast. (**b**) Images of the microwell plate. (i) The microwell plate was customized at two scales, with 1690 microwell array in 72.4 mm diameter area and 151 microwell array in 20.8 mm diameter area having (ii) 600 μm and (iii) 2600 μm depths, respectively. Scale bars are 2 mm. (**c**) Process of spheroid formation in the microwell array. (i) Loading of the HF cell suspension on the microwell array. (ii) Sedimentation of the cells by gravity and guidance by the entrance slope. (iii) Aggregation of the cells at the center of the concave-shaped bottom. (iv) HF spheroid formation in the microwells.

The design of the microwell included a 45° downhill slope (guide wall in Fig. 1b, ii) at the microwell entrance and a concave bottom (Fig. 1b, ii). To pattern the microwell array on the acrylic plate, a computational numerical control (CNC) micro-milling machine (DAVID 3040, David Motion Technology, Incheon, Republic of Korea) was used (supplementary figure S1). In this process, a 90° tapered mill (4STE000900S04, JJTOOLS, Seoul, Republic of Korea) was applied to sculpt the guide wall with a 45° slope. The concave-shaped microwells were then sculpted using a 600 μm diameter ball endmill (2HRBG006160S04, JJTOOLS). Thereafter, an acrylic mold with the microwell array was replicated using polydimethylsiloxane (PDMS, Sylard^®^ 184, Dow, Midland, MI, US) by the double-casting process (Fig. 2a). The PDMS solution was prepared by mixing a prepolymer and a curing agent in a 10:1 ratio before pouring into the acrylic microwell mold. The PDMS solution was cured for 2 h at 80 °C on a hot plate (MSH-30D, DAIHAN Scientific, Wonju, Republic of Korea), and the PDMS master was separated from the acrylic mold (Fig. 2a, i). Before using the PDMS master, the following procedures called double-casting treatment were conducted on the PDMS master: air plasma exposure for 30 s, immersion in ethanol for 10 min, and drying out the ethanol (Fig. 2a, ii). It is known that the double-casting treatment passivates the adhesion-promoting species on the surface of the cured PDMS^16^. Therefore, the PDMS microstructure can be used for casting the PDMS microwell array by double-casting treatment (Fig. 2a, iii). This fabrication process is useful for customizing the number and shapes of the microwells in a microwell plate (Fig. 2b). The microwell arrays were fabricated on the PDMS plate with diameters of 72.4 mm and 20.8 mm; these microwell arrays include 1690 and 151 microwells for mass production of spheroids and image analysis, respectively (Fig. 2b, i). Two types of microwell arrays with depths of 600 μm (Fig. 2b, ii) and 2600 μm (Fig. 2b, iii) were prepared. The diameter of the microwell is 600 μm with the guide wall (45° slope) being installed at the entrance of the microwell, and the bottom has a concave shape.

### Ethical approval

The animal experiments were conducted with ethical approval obtained from the Institutional Animal Care and Use Committee (IACUC) at Sejong University (SJ-20190702), and all protocols related to animal experiments were approved by the IACUC. All experimental procedures were conducted in accordance with the ARRIVE guidelines.

### HF cell preparation

The HFCs used in this study were isolated from plucked HFs obtained from the dorsal skin of 6-week-old mice. First, the mice were cervical dislocated, and 70% alcohol was used to disinfect the back. After disinfection, the dorsal skin (1 cm below the neck to 1 cm above the tail, 2 cm to the left and right sides) was harvested using disposable forceps and scissors before placing in a 10 cm Petri dish containing phosphate-buffered saline (PBS). The skin tissue was cut to a size of 1 × 1 cm and rinsed 2–3 times in 10 cm Petri dish containing PBS while replacing the dish. The well-rinsed skin tissue was then submerged in a mixture of DMEM:F12 (1:1) at a concentration of 2.5 mg/ml Dispase II (D4693, Sigma-Aldrich, St. Louis, MO, USA) (approximately 30 ml) at 4 °C. After overnight treatment, the skin tissue was rinsed with PBS. The HFs were pulled out using forceps and put in DMEM:F12 medium in 60 mm Petri dish. To dissociate the HFCs from HF, the hairs were treated with 10 ml of 0.25% trypsin in a 37 °C water bath. After 5 min, trypsin inactivation was carried out by adding 1 ml of fetal bovine serum (FBS). Then, in a 50 ml tube, the HFs were rinsed with HF culture medium (compositions are listed in supplementary table S1)^17^, and the hair shafts were filtered using a 40 μm mesh strainer. The filtering process was repeated, and the HFCs were resuspended in 1 ml of HF culture medium. The cells were cultured in an incubator with 95% humidity and 5% CO_2_ at 36.5 °C; the HF culture medium was used and replaced every 2 days. The major population of established cells consist of Cd34 and Cd49f. (supplementary figure S2).

### HF spheroid formation

The microwell plates were sterilized using an autoclave (BF-60AC, BioFree CO. LTD, Seoul, Republic of Korea) at 121 ℃ for 15 min. To prevent cell attachment, the microwell plates were precoated by immersion in 4% Pluronic F-127 solution (P2443, Sigma-Aldrich); during this process, air bubbles trapped in the microwells were removed through manual pipetting. After overnight treatment, the Pluronic F-127 solution was washed out twice with D-PBS (Gibco^®^, Thermo Fisher Scientific, Waltham, MA, USA). Before cell loading, the culture medium was poured onto the microwell plate, and the air bubbles trapped in the microwells were blown out by manual pipetting. The cell suspension was prepared to contain approximately 3 × 10^3^ cells in each microwell. The cell suspension was then poured on the microwell plates containing the culture medium (Fig. 2c, i). The cells sank into the microwells by gravity and slid down the slope of the guide wall without cell loss (Fig. 2c, ii). The concave shape of the microwell enabled gathering the cells passively at the center of the bottom (Fig. 2c, iii). One day after cell seeding, the HFCs were aggregated and formed spheroids (Fig. 2c, iv). In the microwell array, the culture media was changed in the 2–3 days. The supplied culture media was filled in the reservoir on the microwell array, and the fresh culture media diffused inside the microwells (supplementary figure S3).

### Bright-field image measurement

The HF spheroids were cultured in microwells and observed using a microscope (CKX41, Olympus, Tokyo, Japan). Using ImageJ 1.53 k (National Institute of Health, Bethesda, MD, US) image processing software, the spheroid outlines were traced manually, and the size and center positions of the spheroids were measured.

### Cell viability analysis

The viability tests of the HF spheroids cultured in the shallow-well and deep-well were analyzed using a LIVE/DEAD^®^ Viability/Cytotoxicity Kit for mammalian cells (Thermo Fisher Scientific). The assay solution contained 0.5 μl of calcein acetoxymethyl ester (calcein AM) solution and 2 μl of ethidium homodimer-1 solution per 1 ml of culture media. The HF spheroids were treated in this solution at room temperature. After 30 min, the HF spheroids were observed using a confocal laser scanning microscope (LSM710, Carl Zeiss, Oberkochen, Germany).

### RNA isolation, cDNA synthesis, and RT-qPCR

RNA of 5 × 10^6^ cells of the HFCs and HF spheroids were extracted with TRIzol™ Reagent (Invitrogen, Thermo Fisher Scientific) according to manufacturer instructions. For reverse transcription, M-MLV Reverse Transcriptase (Invitrogen) was used. Real-time quantitative polymerase chain reaction (RT-qPCR) analysis was performed using the CFX Connect™ Real-Time PCR Detection System (BIO-RAD) with SYBR green real-time PCR master Mix (QPK-201, TOYOBO). PCR was performed by denaturation at 94 °C for 3 min, followed by 40 cycles of annealing at 95 °C for 1 min, 60 °C for 1 min, and 72 °C for 1 min; then, a final extension was performed at 72 °C for 5 min. The primer sequences are shown in supplementary table S2. The expression levels were normalized to those of endogenous *Gapdh,* and the data were analyzed using the delta-delta C_t_ method.

### Immunocytochemistry of HF spheroids

3D-cultured HFCs were fixed with 4% paraformaldehyde overnight at 4 °C. After washing with PBS, the HF spheroids were embedded with FSC22 frozen section media (3,801,480, Leica Biosystems, Wetzlar, Germany). Cryofixed molds were cut into 10–20 µm thickness using a microtome (HM525, Thermo Fisher Scientific). After attachment to silane-coated slide glass (5116-20F, Muto Pure Chemicals, Tokyo, Japan), the samples were fixed with 4% paraformaldehyde, washed with PBS, and stored at − 80 °C.

For immunocytochemistry, HFCs were placed on 12 mm round cover glass in a 12-well culture and fixed with 4% paraformaldehyde. Then, treatment was performed with 1% sodium dodecyl sulfate (SDS) in PBS for 1 min for antigen retrieval. After blocking with 3% horse serum in 0.1% PBST for 1 h, the samples were incubated with primary antibodies overnight at 4 °C. The cells were washed with 0.1% PBST 3 times. The secondary antibodies were used in a 1:500 dilution factor for 1 h. After washing with 0.1% PBST thrice, the secondary antibodies were treated at room temperature for 1 h. After washing, 0.1% Hoechst was applied as treatment for 3 min. The slides were subsequently mounted on a fluorescence mounting medium (Vectashield, Vector Laboratories, Newark, CA, US).

### Fluid dynamics analysis in microwell

**Figure 3:**
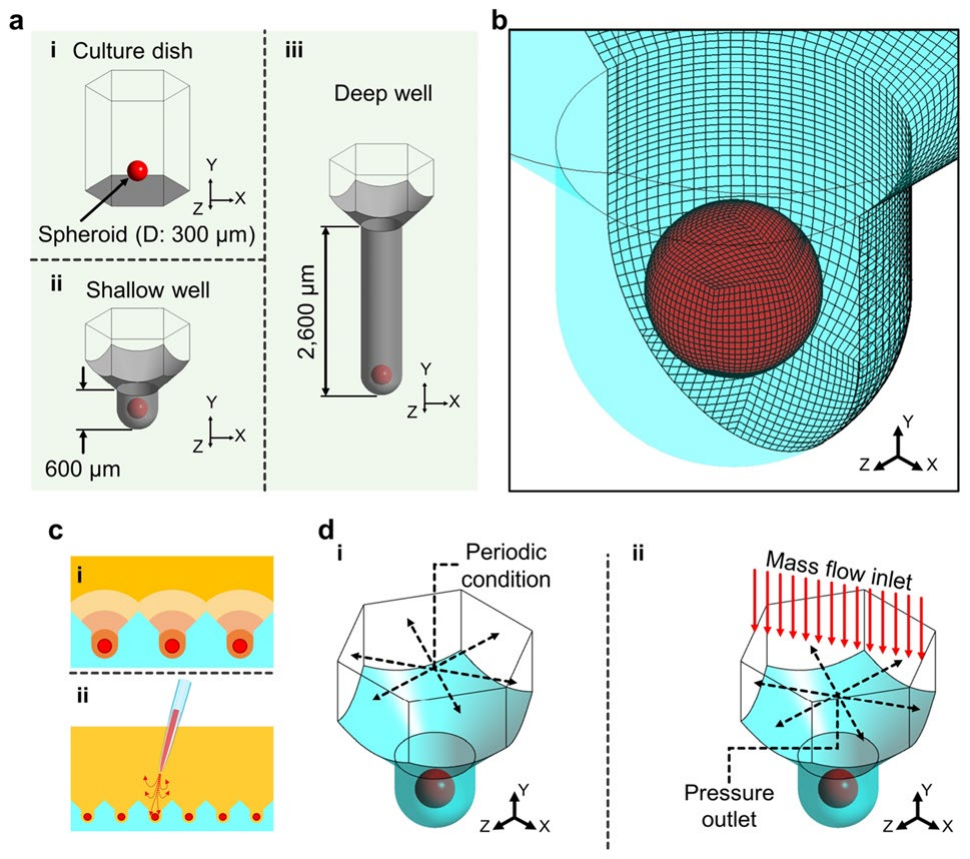
Computational models and conditions. (**a**) Computational geometries of the (i) culture dish, (ii) shallow well, and (iii) deep well. (**b**) Computational grid system. (**c**) Phenomena of exposing the spheroids in the microwell array. (i) Diffusion of biomolecules secreted from the spheroids. (ii) Convective flow generated during spheroid culture. (**d**) Simulated boundary conditions for the (i) diffusion of biomolecules and (ii) convective flow in the microwell.

Computational simulations were conducted to investigate the flow phenomena around the spheroid according to microwell depth using the commercial CFD tool ANSYS Fluent 19.2 (ANSYS, Inc., Canonsburg, PA, US). To simplify calculations for the entire microwell array, a single microwell including a single spheroid was adopted as the 3D computational geometry (Fig. 3). The flow dynamic environment of the conventional culture dish was compared with that of the microwell, and the spheroid diameter was assumed to be 300 μm (Fig. 3a, i). The computational geometries were constructed with depths of 600 μm for the shallow microwell and 2600 μm for the deep microwell, respectively (Fig. 3a, ii and iii). The hexahedral grid was constructed with 162,000 elements for the shallow microwell, and the same grid density was applied to the culture dish and deep microwell model (Fig. 3b). The grid density was determined by the grid independence test with 48,600- and 385,200-element grid models of the shallow microwell geometry (supplementary figure S4). In the microwell array, each spheroid is influenced by biomolecules, such as nutrients, cellular biomolecules, and waste products, and their diffusions contribute to the interactions with neighboring spheroids (Fig. 2c, i). In addition, during the cell culture experiments, operational handling and pipetting generate convective flows inevitably, and the spheroids in the microwell are exposed to this flow directly (Fig. 2c, ii). To predict the effects of biomolecular diffusion and convective flow, the boundary conditions were set for each phenomenon (Fig. 2d). In the diffusion simulation model, the periodic condition was applied to each of the opposite sides to consider the effects of spheroids in the six neighboring microwells (Fig. 2d, i). The species transport model was applied to assume diffusion of the biomolecules from the spheroid with 50% mass fraction, and the diffusion coefficient was 10^–10^ m^2^/s^18^. The species transport model predicts the mass fraction without source and chemical reactions using the following conservation equation (Eq. 1), and for laminar flow, the diffusion flux is determined based on Fick’s law without thermal diffusion (Eq. 2)^19^.

1 $$\frac{\partial }{\partial t}\left(\rho {Y}_{i}\right)+\nabla \cdot \left(\rho \overrightarrow{v}{Y}_{i}\right)=\nabla \cdot {\overrightarrow{J}}_{i}$$

2 $${\overrightarrow{J}}_{i}=-\rho {D}_{i,m}\nabla {Y}_{i}$$

where *ρ* is the fluid density, *Y*_*i*_ is the local mass fraction of each species, $\overrightarrow{v}$ is the velocity component, ${\overrightarrow{J}}_{i}$ is the diffusion flux of each species, and *D*_*i,m*_ is the mass diffusion coefficient of species *i*. The culture medium was assumed as a homogeneous and incompressible Newtonian fluid, i.e., water of 998.2 kg m^−3^ density^10^. To observe the diffusion process, transient simulations were performed, and the timestep was set to 10 s, with each timestep being calculated for 500 iterations. The convective flow analysis considered the pipetting flow generated during the cell culture process. The flow direction was assumed to be perpendicular to the microwell, and a mass flow rate of 1 ml/s was set for the top of the geometry (Fig. 2d, ii). The pressure outlet condition (1 atm) was applied to the boundary with adjacent microwells. Laminar flow was simulated at steady state following the governing equations of continuity (Eq. 3) and momentum (Eq. 4)^19^.

3 $$\frac{\partial \rho }{\partial t}+\nabla \cdot \left(\rho \overrightarrow{v}\right)={S}_{m}$$

4 $$\frac{\partial (\rho \overrightarrow{v})}{\partial t}+\nabla \cdot \left(\rho \overrightarrow{v}\overrightarrow{v}\right)=-\nabla \rho +\nabla \cdot \left(\tau \right)+\rho g+F$$

where *S*_*m*_ is the mass added from source, *τ* is the stress component, *g* is the gravitational acceleration, and *F* is the force component. Each case was calculated for 10,000 iterations, and the residuals of all cases were less than 1 × 10^–6^.

## Results and discussion

### Expression of cell markers in HF spheroids

**Figure 4:**
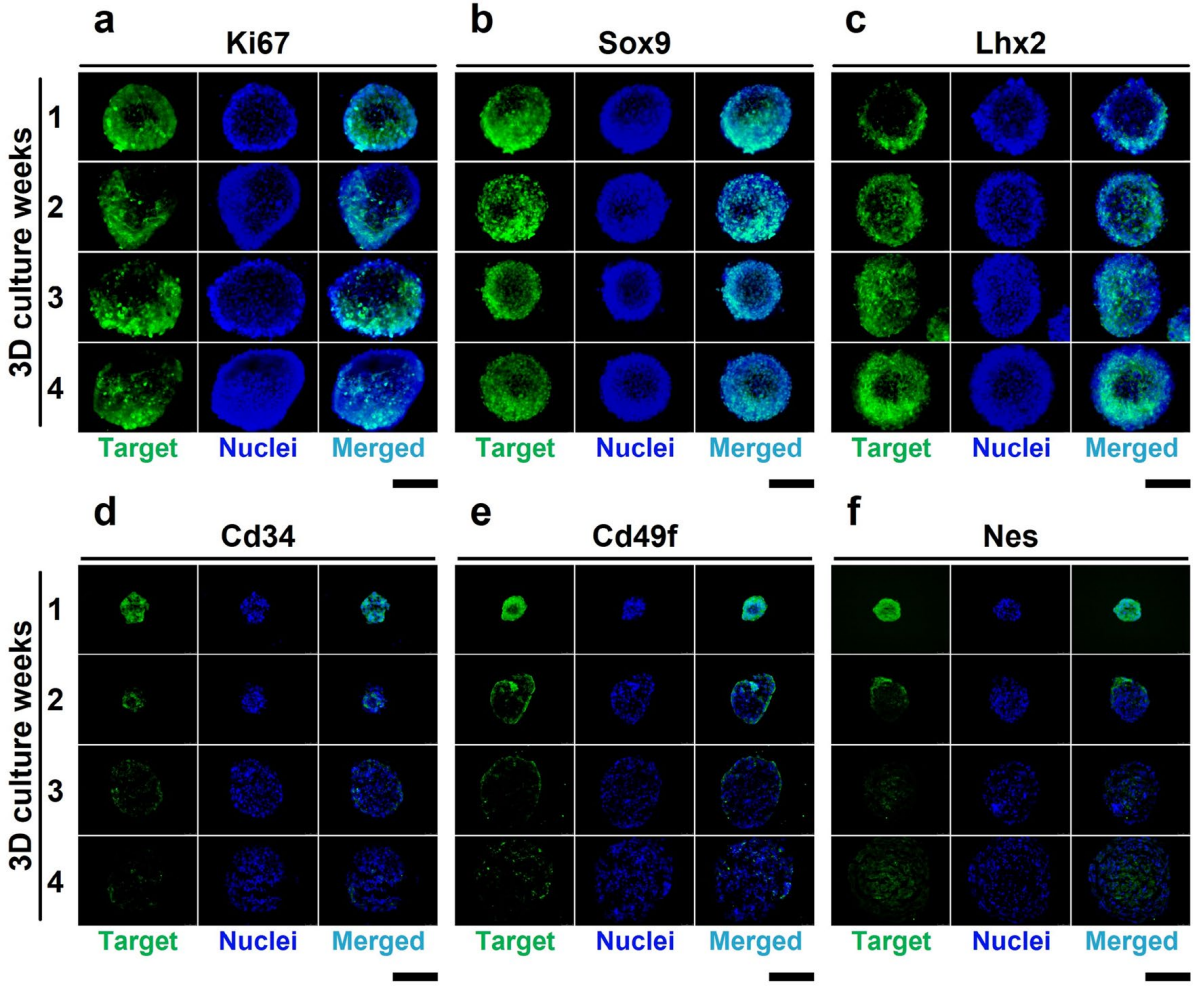
Protein expressions in spheroid-cultured HFCs. (**a**) Immunohistochemistry (IHC) of Ki67 as a marker of cell proliferation by using cryo-section of spheroids. (**b**, **c**) IHCs of Sox9 and Lhx2 as markers of key transcription factors of bulge stem cells. (**d**–**f**) IHCs of Cd34, Cd49f., and Nestin as markers of bulge stem cells. (Color indicators; green: target gene, blue: nuclei (Hoechst), blue-green: merged signals. Scale bars are 200 μm).

Different cell types express their own marker genes. In HFs, the expressions of both Cd34 and Cd49f. are known as bulge stem cell markers^20,21^. For additional cell identification, the isolated cells were tested with antibodies against Krt14, Krt10, Sox9, and Vimentin (supplementary figure S5). The positive expression of Krt14 may indicate that the cells are epithelial, in correlation with bulge-specific markers Cd34 and Cd49f^22^. In contrast, the absence of Krt10 expression might suggest that external IFE cells were not included^23^. Sox9 is a transcription factor of bulge cells. Vimentin, as a marker of fibroblast, was not detected in the isolated cells. Cd34/Cd49f.-positive bulge stem cells have proliferative and differentiation potentials to maintain HF structure during the hair cycle^24,25^. To observe cell proliferation during HF spheroid cultivation, the immunocytochemistry (ICC) of Ki67 was performed. The ICC of Ki67, a cell proliferation marker, demonstrates that cell division occurs at both the inside and surface of the HF spheroid (Fig. 4a). From this result, we monitor that proliferative potential of the HF spheroids was maintained for 4 weeks. For further identification of the HF spheroids in the microwell, we observed key transcriptional factors of the bulge stem cells, such as Sox9 and Lhx2 (Fig. 4b,c). Consistent expression of Sox9 and Lhx2 may indicate that the HF spheroids maintain their identity as bulge stem cells. Further, early HF spheroids express the surface markers of bulge stem cells, such as Cd34, Cd49f., and Nestin (Fig. 4d–f). Surface marker expressions could imply that early microwells provide similar environment for hair bulge. However, the expressions of surface markers of the bulge stem cells diminish after 4 weeks. The IHC data show that the expressions of Sox9 and Lhx2 are maintained but that the Cd34, Cd49f., and Nes expressions are downregulated. From the alteration of the protein marker expressions, we hypothesize that the long-term culture of bulge stem cells in microwells may not only decrease marker expression, but also increase other gene expression.

### Activation of Gli1 gene in HF spheroids

**Figure 5:**
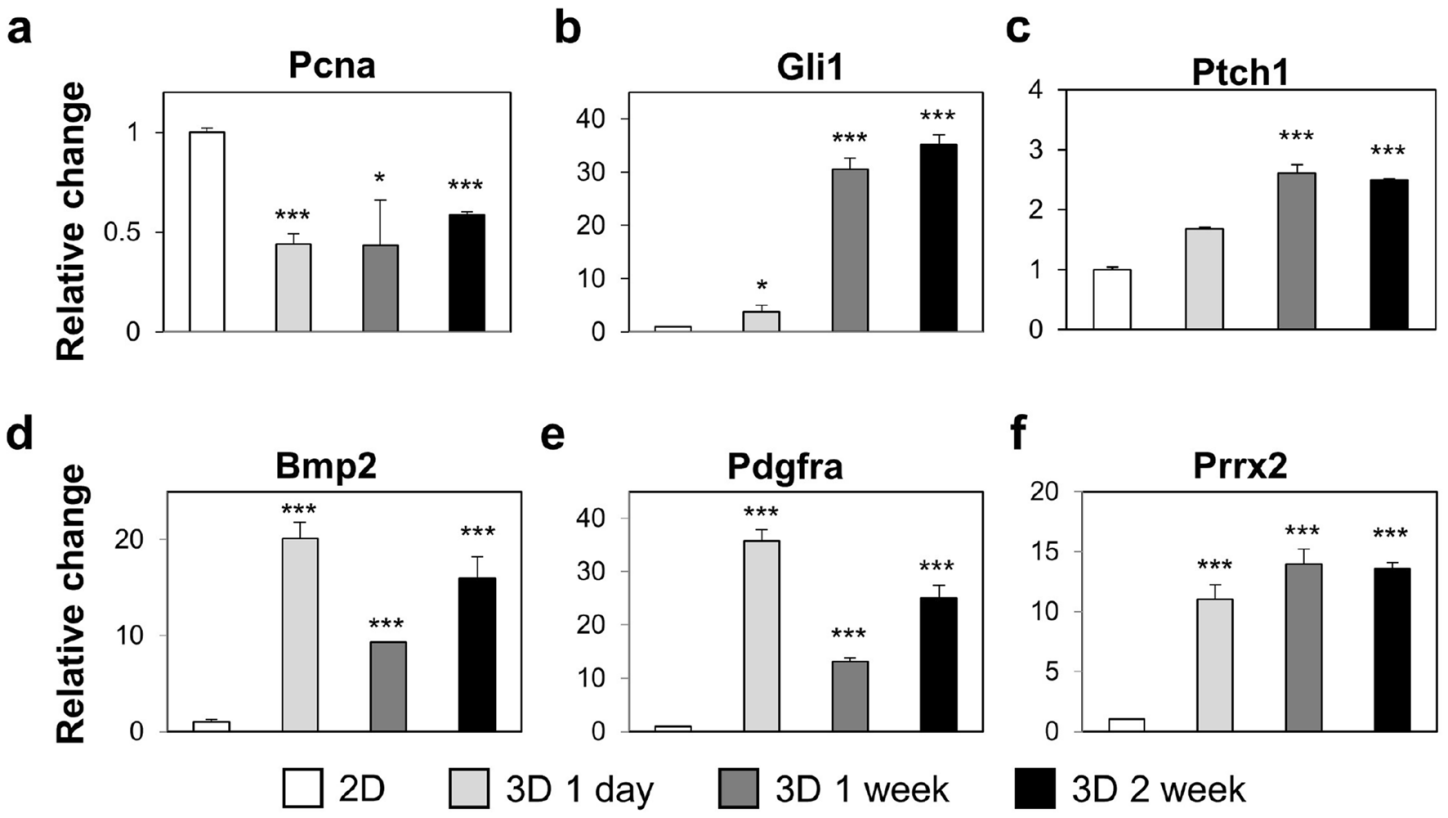
Altered mRNA expressions in spheroid-cultured HFCs. (**a**) mRNA expression of Pcna as a proliferation marker. (**b**, **c**) Increased mRNA expression of the Shh-pathway-related genes Gli1 and Ptch1 by spheroid culture. (**d**–**f**) mRNA expressions of Bmp2, Pdgfra, and Prrx2 as downstream target genes of the Shh pathway. (Asterisk means *p*-value; * indicates *p* < 0.05, *** indicates *p* < 0.001).

Bulge stem cells have diverse role in HFs, such as construction of hair bulge, inner root sheath, outer root sheath, and sebaceous gland cells^26^. Above all, the Gli1-expressing cells from the bulge are a key population to maintain and regenerate hair follicular epidermis^27–30^. We first confirmed gene expression for Pcna, which is a cell cycle marker during DNA division (Fig. 5a). The expression level of Pcna was down-regulated compared to the 2D environment. While HF spheroids were proliferative, the proliferative capacity possibly decreased compared to 2D environment. Next, we confirmed highly upregulated Gli1 expression after 2 weeks of cultivation of the HF spheroids (Fig. 5b). As the Gli1 expression is dependent on Sonic hedgehog (Shh) signaling, the expression of Ptch1, a receptor of Shh, was also confirmed in the HF spheroids (Fig. 5c). From the increased mRNA expressions of Gli1 and Ptch1, the culture of HFCs in the microwell to form spheroids would activate Shh signaling within the spheroid-forming cells. To investigate the effects of Gli1 expression in HF spheroids, downstream signals of Gli1 in the bulge stem cells, such as Bmp, Pdgfra, or Prrx2, were observed by RT-qPCR (Fig. 5d–f). These results mean that Gli1may activate bulge stem cells by transcriptional regulation in microwell environments. It has been known that the 3D cellular microenvironment implemented in the spheroid mimics the cell responses to drugs more similarly to the in vivo environment than the 2D environment^31^. Therefore, it is anticipated that this HF spheroid culture methodology will be used in the development of drugs for HF regeneration, which is also a part of our future research plan.

### Influence of microwell depth

**Figure 6:**
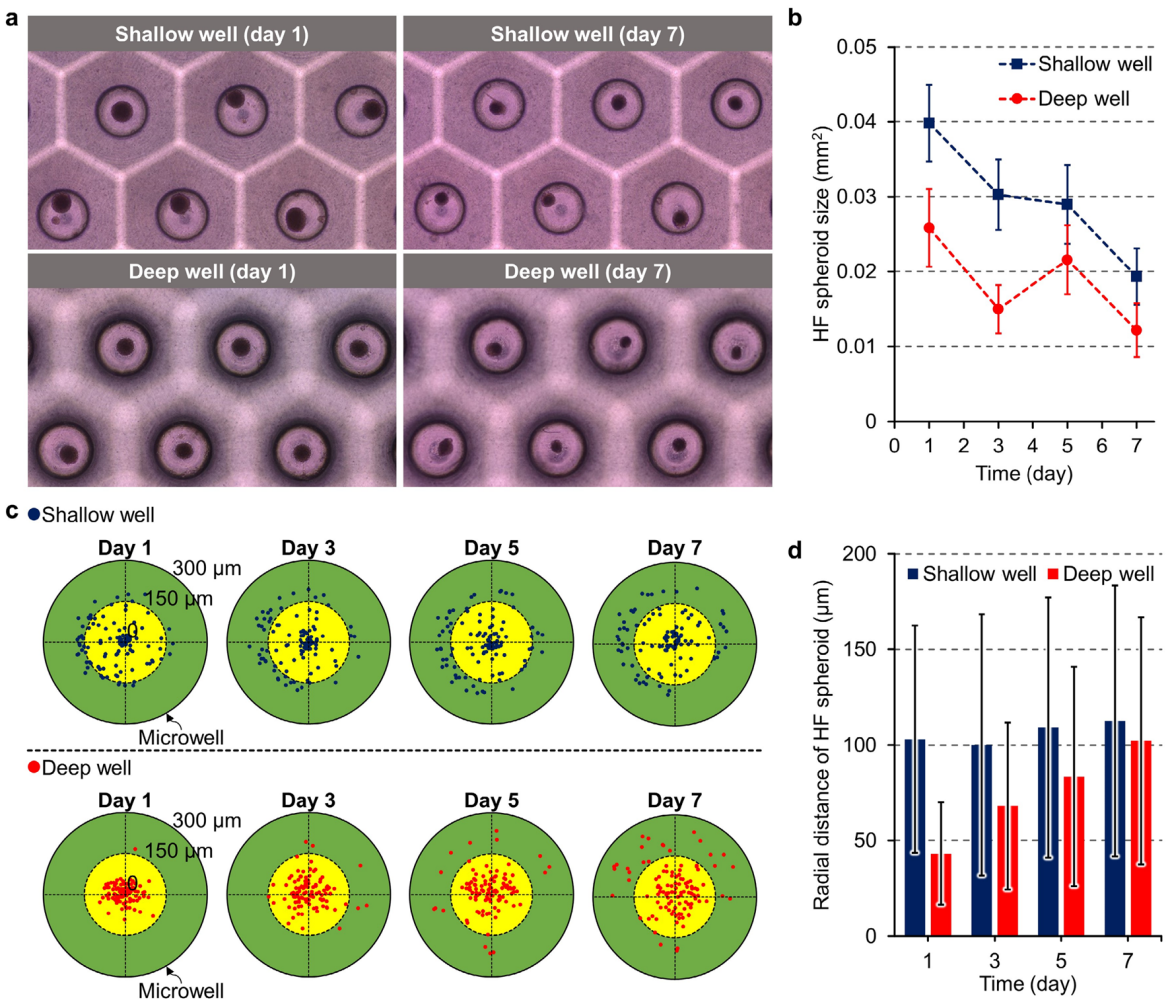
Influence of microwell depth on HF spheroid formation. (**a**) Images of HF spheroids cultured in shallow and deep wells. (**b**) Sizes of the HF spheroids cultured in the shallow and deep wells from days 1–7 (error bars are standard deviation). (**c**) Dot plot showing the center position of HF spheroids in the shallow (blue dots) and deep wells (red dots). (**d**) Radial distances of the HF spheroids from the microwell center in the shallow and deep wells (error bars are standard deviation).

Through the live and dead assay, the cells survival inside the HF spheroids were observed regardless of the microwell depth, and necrosis of cells was confirmed in the center of the spheroid (the confocal images are shown in supplementary figure S6). To confirm the effect of microwell depth on HF spheroid formation, bright field images of HF spheroids cultured in microwells were analyzed. The shape of the microwell has a direct impact on the formation and morphology of spheroids, and the depth of the microwells is considered as a major geometric parameter. To investigate the effects of microwell depth, two types of microwell arrays with 600 μm (shallow well) and 2600 μm (deep well) depths were fabricated (Fig. 2b, ii and iii) and used to culture and observe the HF spheroids for 1 week (Fig. 6). The cells loaded into the microwell aggregated at the bottom over a period of 24 h (Fig. 6a). One day after cell loading, HF spheroids were successfully formed in both types of microwell arrays; however, the HF spheroids differed in size and position in the microwell depending on depth. The HF spheroids formed in the deep well were smaller than those formed in the shallow well. For 7 days, in both the shallow and deep wells, the sizes of the HF spheroids were reduced, and the HF spheroids cultured in the deep well were found to be smaller than those in the shallow well, which was consistent with the tendency observed on day 1. The sizes of the HF spheroids in the shallow and deep wells were measured every 2 days and quantitatively compared (Fig. 6b). The number of HF spheroids used in the measurement was 109 for each microwell type (images used for measurements are shown in supplementary figure S7). On day 1, the average sizes of the HF spheroids were 0.040 mm^2^ and 0.026 mm^2^ for the shallow and deep wells, respectively. In the shallow well, the overall size reduction of the spheroids was observed over days 3–5, and a tendency for the spheroids to be maintained was confirmed (0.030 mm^2^ on day 3 and 0.029 mm^2^ on day 5); after 7 days of cultivation, the average size of the spheroids was measured to be 0.019 mm^2^. In the deep well for 7 days, the sizes of the spheroids decreased by 0.012 mm^2^; however, the sizes of the spheroids measured on day 3 (0.015 mm^2^) and day 5 (0.022 mm^2^) temporarily increased. The standard deviation of the spheroid size was 0.0052 mm^2^ or less for all cases.

Meanwhile, the distribution of the HF spheroid position was also impacted by the microwell depth. The center positions of the spheroids were measured for 1 week (Fig. 6c, the images used in the measurements are shown in supplementary figure S7). On day 1, in the deep well, almost all HF spheroids were aligned at the central region inside a 150 μm radius (yellow-colored area in Fig. 6c), whereas in the shallow well, the spheroids were frequently located outside a 150 μm radius (green-colored area in Fig. 6c). The spheroids confirmed on day 1 show the situation right after cell aggregation (process shown in Fig. 2c); therefore, the spheroids located near the wall indicate that the cells were affected by external forces during spheroid formation. Handling by the researchers, such as pipetting for changing the culture media and transport of the system within the laboratory for observing and treating the samples, are inevitable for experiments using microwell arrays, which are accompanied by external forces that affect the cells and spheroids in the microwell arrays. For one week, the distribution of the HF spheroids gradually expanded from the center to the periphery in the microwells regardless of microwell depth. However, the deep well inhibited movement of the HF spheroids on the microwell bottom compared to the shallow well. The average radial distances of the HF spheroids cultured in the shallow and deep wells from the center were compared (Fig. 6d). In the shallow well, the HF spheroids were located at a distance of more than 100 μm on average over a week and tended to slightly disperse by 112.6 μm as of day 7. On the other hand, in the deep well, the radial distances of the HF spheroids were measured to be smaller than those in the shallow well for a week, and the HF spheroids located at an average distance of 43.1 μm on day 1 were dispersed by 102.3 μm as of day 7. The standard deviations, indicated by the error bars in the graphs, provide a quantitative measure of the dispersion of the HF spheroids (Fig. 6d). The distribution of the HF spheroids spread over time regardless of the microwell depth, and in the deep well, the distribution of the spheroids was uniformed compared to the shallow well. Especially, a significantly lower standard deviation was confirmed in the deep well on day 1, indicating that the HF spheroids were uniformly positioned at the microwell center in the process of spheroids formation and at the beginning of cultivation.

**Figure 7:**
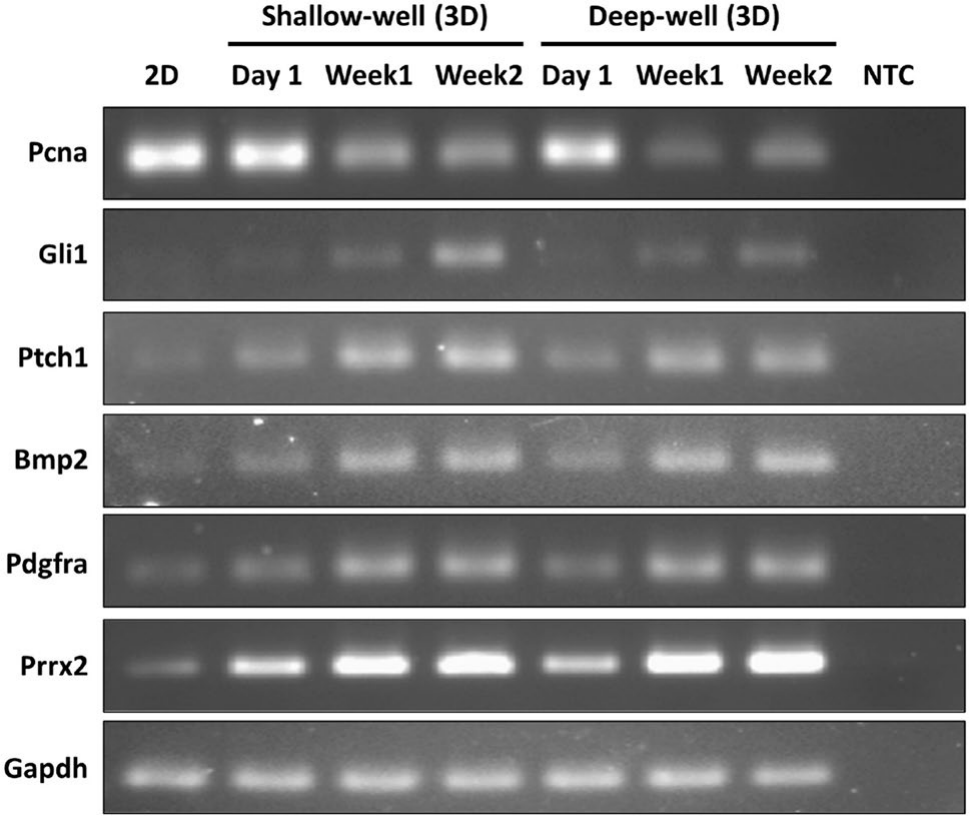
Reverse transcription-PCR to compare mRNA expressions of the spheroids in the shallow and deep microwells.

Additionally, we compared the gene expressions of Pcna, Gli1, Ptch1, Bmp2, Pdgfra, and Prrx2 in the shallow and deep wells (Fig. 7, supplementary figure S8). The Shh-pathway-related genes were commonly upregulated in both the shallow and deep wells over time compared with 2D cultured HFCs; therefore, at the biological level, 3D microenvironment of the microwell may possibly activate the Shh pathway of HFCs. Furthermore, Gli1 expression was shown to be much higher in shallow microwell HF spheroids than in deep microwell HF spheroids. Since Gli1 expression is attributed to the binding of Shh to Ptch1 on the cell surface, activation of the Gli1 signaling pathway in the deep well may be delayed compared with that in the shallow well.

### Fluid dynamics in the microwell

**Figure 8:**
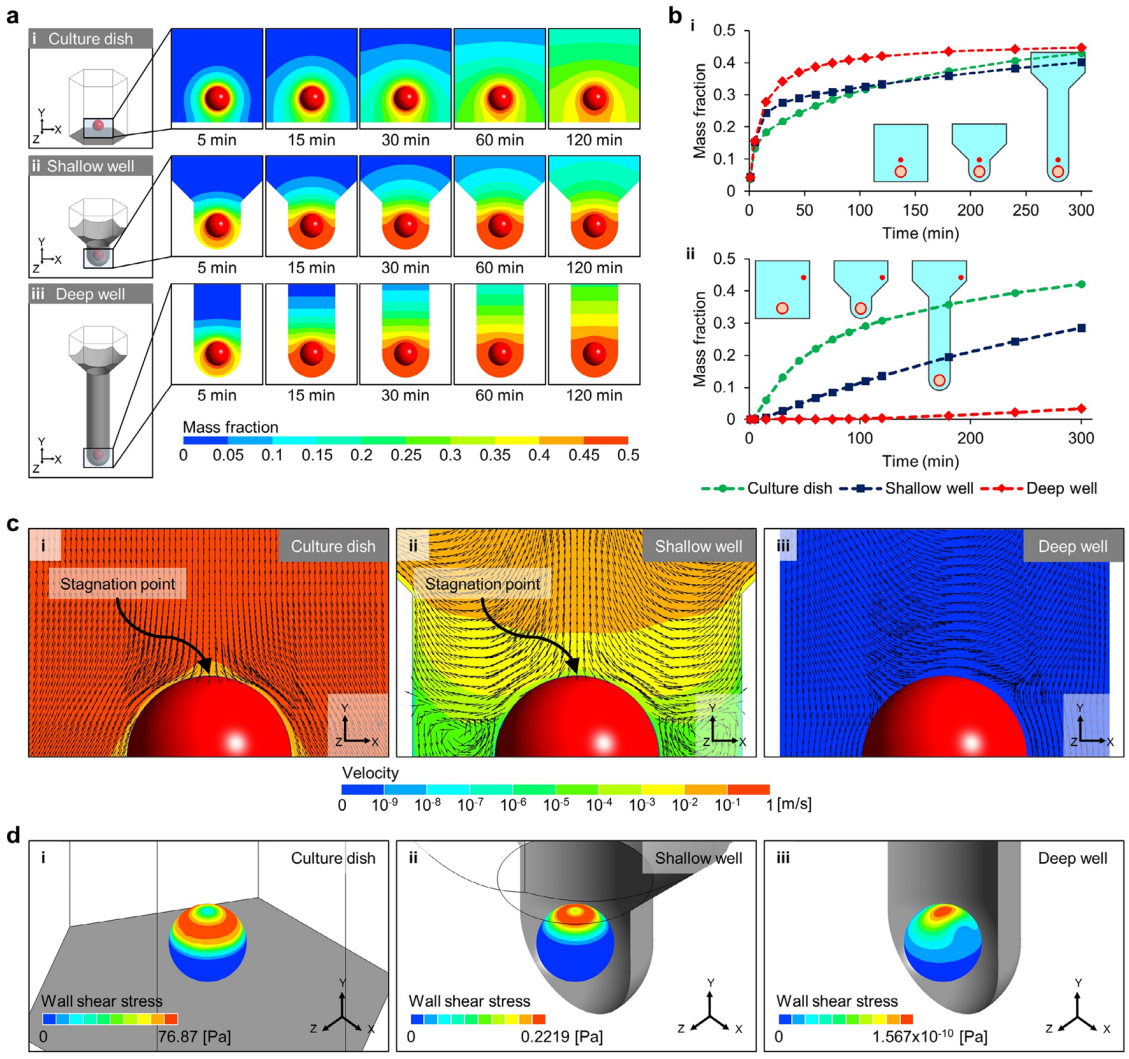
Computational prediction of the flow phenomena around HF spheroids cultured in a culture dish as well as shallow and deep microwells. (**a**) Mass fraction contours of the spheroid molecular diffusion (i) on the culture dish and in the (ii) shallow and (iii) deep wells. (**b**) Graphs of spheroid molecular diffusion at the (i) microwell entrance and (ii) boundary of the computational domain. The mass fraction values are predicted at the red points of the inset figures, respectively. (**c**) Velocity contour and normalized velocity vector of the convective flow applied to the spheroids (i) on the culture dish and in the (ii) shallow and (iii) deep wells. (**b**) Shear stress contours on the spheroids formed by convective flow (i) on the culture dish and in the (ii) shallow and (iii) deep wells.

The microwell depth is a structural parameter that determines the hydrodynamic environment around the spheroids in the microwell. The flow phenomenon in the microscopic space in the microwell was analyzed using CFD, and to investigate the effect of the microwell, the model simulating the spheroids cultured in the culture dish assumed an environment was compared with the microwell models (Fig. 8). The diffusion of biomolecules secreted by the spheroids was suppressed in the micro-space of the microwell (Fig. 8a). Physical barriers, such as the wall and bottom of the microwell, prevent diffusion of biomolecules generated by the spheroids, which causes concentration of these components in the vicinity of the HF spheroids. In the microwell, the entrance that is exposed to the external environment was a channel for biomolecular diffusion, whereas in the culture dish, diffusion occurred in all directions except the bottom. In the shallow well, the microwell entrance was close to the spheroid; therefore, the biomolecules of the spheroids spread out more easily compared with those in the deep well for 120 min. Consequently, around the HF spheroids in the deep well, the highest mass fraction of biomolecules was formed. In each model, the mass fraction was measured at a point 150 μm above the spheroids (red point in the inset of Fig. 8b, i). For all cases, the mass fractions increased rapidly within 50 min. In the deep well, the increase rate of the mass fraction was steepest, and the highest maximum value (0.447) was measured; in the culture dish, the lowest increase rate was measured. These simulation models considered the molecular diffusion of neighboring spheroids by applying periodic conditions. The maximum mass fraction measured in the culture dish (0.430) was larger than that in the shallow well (0.401), implying that the effects of neighboring spheroids were more prominent over time in the culture dish. The effects of the neighboring spheroids on the microwell depth were elucidated through measurements taken at a point near the boundary of the computational domain (red point in the inset of Fig. 8b, ii). At this point, the tendency of the mass fraction showed that the biomolecular interactions between the spheroids in the microwell were more blocked than in the culture dish. In the deep well, since the spheroids were the most isolated from the external environment, interactions with neighboring spheroids were difficult compared to the other cases. In previous studies, it has been reported that the limitation of nutrient supply and difficulty of biochemical signaling from the cells in external area due to isolation of the spheroids in the microwells can affect the state of the spheroids^32^. In the present study, it was also confirmed by CFD analysis that the interactions between the inside of the microwell and external environment decreased as the microwell depth increased (Fig. 8a,b); further, it was experimentally observed that the spheroid size was smaller in the deep well (Fig. 6a,b). Although more detailed verifications are needed, this reveals that the culture environment according to microwell depth regulates the activation period of the Shh signal pathway in the HF spheroids (Fig. 7); this finding serves as evidence for the feasibility of our hypothesis that the geometric parameters of the microwell induce biological alterations in the HF spheroids.

In the convective flow simulation, pipetting flow with a mass flow rate of 1 ml/s applied perpendicular to the spheroid was assumed (Fig. 3d, ii). This simulation condition represented the internal flow applied to the spheroids in the microwell system generated during experimental procedures, such as pipetting and handling of the microwell array. In the microwell, although the flow was transferred to the spheroid, the effects of the flow were considerably attenuated, whereas in the culture dish, the flow was directly applied to the spheroid without structural obstruction (Fig. 8c, i and ii). Specifically, the deep well completely protected the spheroids from convective flow (Fig. 8c, iii). The velocity vector in the culture dish and shallow well formed the stagnation point on the spheroid top surface, which implies that the spheroid in the shallow well was exposed partially to the vertically applied flow (Fig. 8c, i and ii). However, in the deep well, a velocity vector field with a completely different pattern was generated (Fig. 8c, iii), and the flow velocity contour around the spheroid was also predicted to be low enough to ignore (< 10^–9^ m/s). The flow generated wall shear stress in the upper part of the spheroids by the flow (Fig. 8d). In the culture dish and shallow well, a low-stress region was formed at the center of the wall shear stress area owing to the decrease in flow velocity in the stagnation region (Fig. 8d, i and ii). In the culture dish, the maximum wall shear stress was measured as 76.87 Pa, whereas in the shallow well, it was calculated to be 0.2219 Pa, confirming that the effects of the flow were reduced by the microwell. On the other hand, no stagnation region was identified in the spheroids of the deep well, and the maximum wall shear stress was calculated as 1.567 × 10^–10^ Pa, which is negligibly low compared to other cases (Fig. 8d, iii). As a result, it was confirmed that the microwell functions to protect the spheroids from external flow, and for greater depths, more complete protection is possible, which is related to the experimental results on the position of the spheroid in the microwell (Fig. 6c,d).

The experimental results shown above confirmed that the deeper the microwell, the smaller the HF spheroids were formed, and the HF spheroids were aligned to the center of the microwell. These phenomena were analyzed by CFD simulation on the diffusion and flow inside microwells. The depth of the microwell reduced the effect of convection flow on the spheroid and hindered the diffusion phenomenon in the microwell. Therefore, in order to precisely control the spheroid cultured in the microwell array and create an environment suitable for growth, a detailed investigation of the optimized microwell depth is required. Based on previous studies^33,34^ that highlighted shear stress and molecular concentration as factors inducing cell responses, it is anticipated that both HFCs and HF spheroids will respond to flow stimulation, and the microwell depth could potentially play a crucial role in controlling this phenomenon. Therefore, additional research is needed, incorporating delicately refined geometric definitions and microscopic cell monitoring. Additionally, researchers must recognize the significance of various geometrical parameters, especially depth, as they may exert mechanical and biological effects on spheroids in experiments employing microwell platforms.

## Conclusion

To investigate the effects of the 3D cellular environment on HF regeneration and to inform the necessity for utilizing heterogenous HFCs, we conducted experiments on HF spheroids consisting of several type of cells using the microwell array. Compared to 2D cell culture conditions, the 3D microenvironment of spheroids reduced proliferation of the HF-derived cells; however, the expressions of Shh transducers, such as Gli1, Ptch1, Bmp, Pdgfra, and Prrx2, were promoted more. During the 4 weeks, HF spheroids maintain the cell proliferation ability and continuously express Sox9 and Lhx2, which are key transcription factors of bulge stem cell, while Cd34, Cd49f., and Nestin expressions decrease. These results suggest that the 3D culture conditions of the microwell are crucial external factor for HF-derived cells to regulate transcription of gene related to HF morphogenesis. However, despite of Gli1 induction by our microwell system, the single sort of cellular population had limitation to observe further developmental features. This is because Gli1 positive cells of hair follicle, which located in upper bulge or isthmus, were known as their function of anagen regeneration or epidermal regeneration during wound healing^27–29^. To overcome this conditional obstacle, transplantation would mimic regenerative roles of Gli1-positive cells related to hair follicle or epidermis^23,35,36^. Additionally, the effects of microwell depth on the HF spheroids were investigated. As a result, the biological effects by the microwell depth were observed at the early stages of spheroid cultivation. The mechanical influences were confirmed by CFD analysis. The deep microwell extremely isolated the spheroids from the external environment, which reduced molecular diffusion within the microwell and rendered the external flow influences inactive. These analyzes imply that the mechanical condition modified by the geometric parameters of the microwell, including the depth, regulates the state of the HF spheroid. Therefore, the microwell system may provide more accurate conditions to recreate the environment for hair regeneration or growth. Progressed techniques of 3d culture in the future would be a sophisticated and effective system for hair follicle research.

HF is significantly limited in amount in the human body; therefore, the efficient utilization of HF derivatives in experiments should be emphasized. This study successfully verified the feasibility of HF research using all types of cells derived from HF tissues. For future research aimed at HF regeneration for drug development and improvement of transplantation rates, further intensification of research on the 3D culture conditions of HFCs, along with the optimal design of microwell arrays, is warranted.

### Supplementary Information

Supplementary Information.

## Data Availability

Original data are available from the corresponding author upon reasonable request.
