# Supplementary material for: Biological and mechanical influence of three-dimensional microenvironment formed in microwell on multicellular spheroids composed of heterogeneous hair follicle stem cells
Source: Sci Rep. 2023 Dec 20;13:22742. doi: 10.1038/s41598-023-49510-6 (PMC10733424; doi:10.1038/s41598-023-49510-6)
Supplement: Supplementary file 1 — Supplementary Information. [file 41598_2023_49510_MOESM1_ESM.pdf]

Supplementary Information

Biological and mechanical influence of three-dimensional microenvironment formed in microwell on multicellular spheroids composed of heterogeneous hair follicle stem cells

Seungjin Lee[1,5], Nackhyoung Kim[2,5], Sung-Hwan Kim[3], Soo-Jong Um[2]* and Joong Yull Park[1,4]*

[1] Department of Mechanical Engineering, Graduate School, Chung-Ang University, 84 Heukseok-ro, Dongjak-gu, Seoul 06974, Republic of Korea
[2] Department of Integrative Bioscience and Biotechnology, Sejong University, 209 Neungdong-ro, Gwangjin-gu, Seoul 05006, Republic of Korea
[3] Cellsmith Inc., 38 Pungseong-ro, Gangdong-gu, Seoul 05393, Republic of Korea.
[4] Department of Intelligent Energy and Industry, Graduate School, Chung-Ang University, Seoul 06974, Republic of Korea

[5] Seungjin Lee and Nackhyoung Kim contributed equally to this work.
* Joong Yull Park and Soo-Jong Um contributed equally to this work as co-corresponding authors.

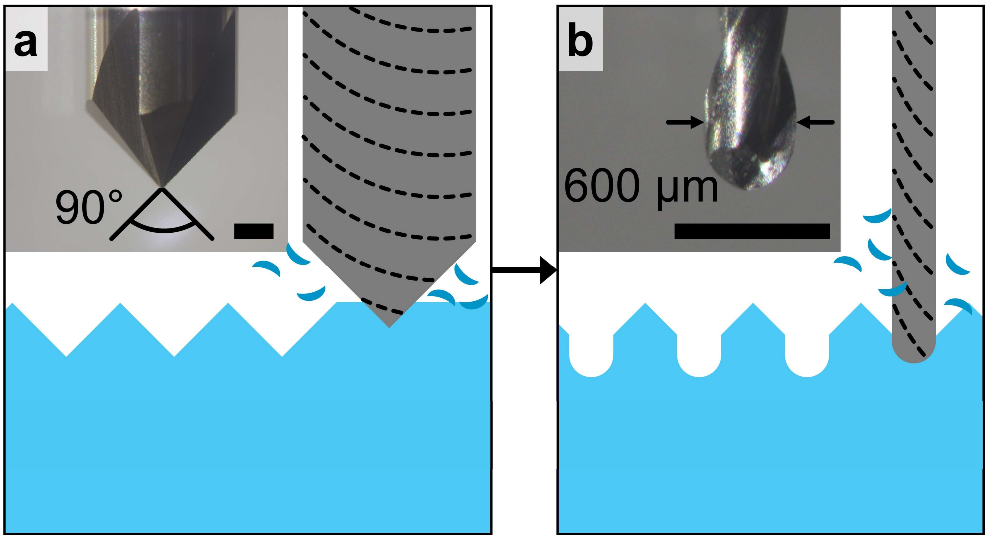

Figure S1. Machining tool for the acrylic microwell mold in the CNC micro-milling process. (i) Funnel-shaped guide wall is cut using a 90° tapered mill, and (ii) concave-shaped microwells are carved with a 600-μm-diameter ball endmill. Scale bars are 1,000 μm.

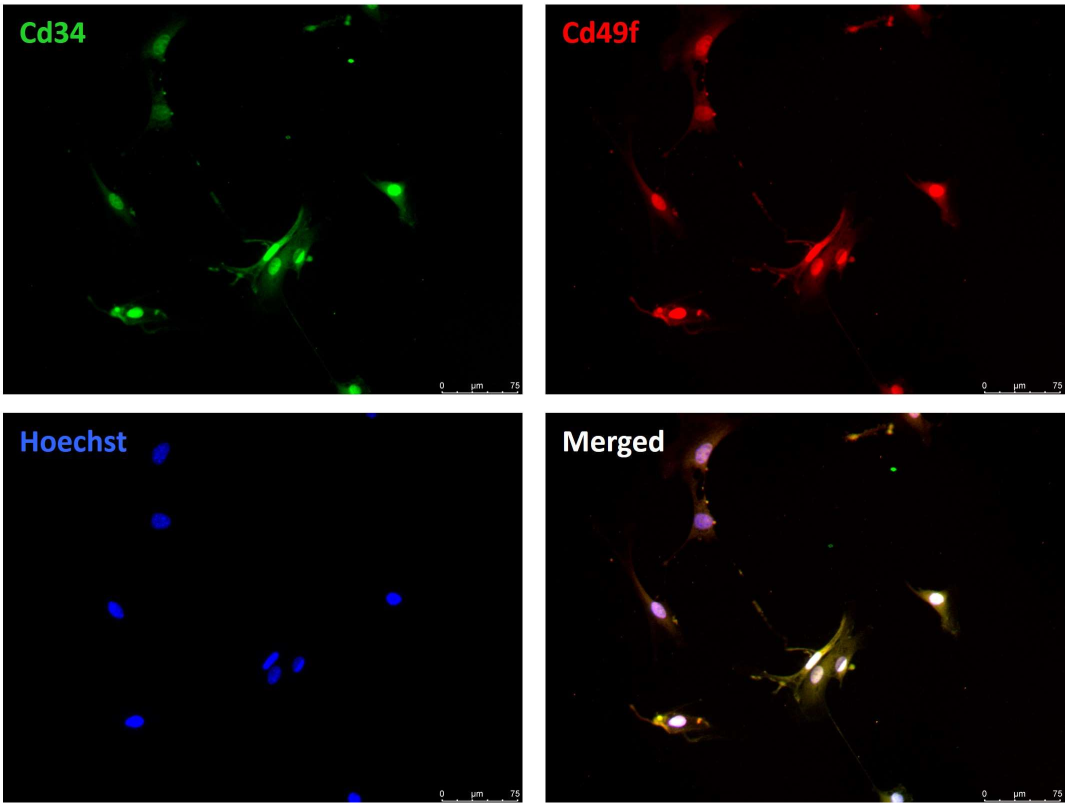

Figure S2. Immunostaining of HFCs against Cd34 and Cd49f.

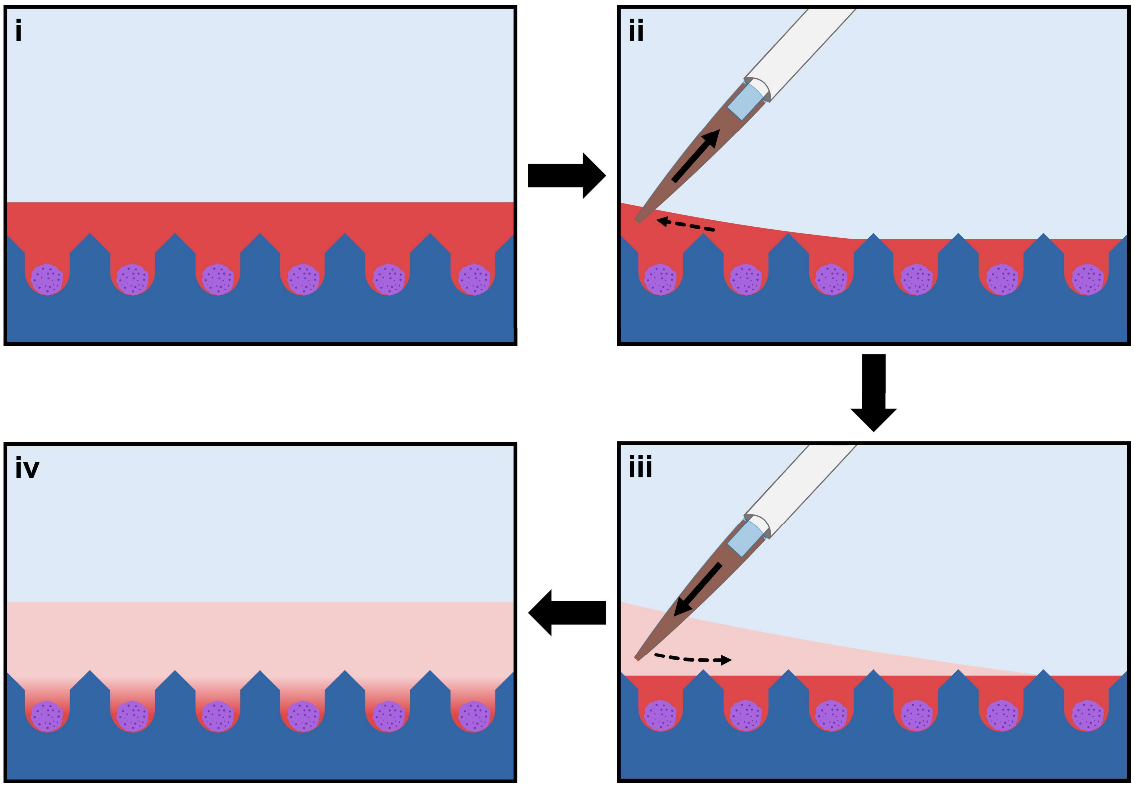

Figure S3 Culture media solution replacement process. (i) Situations where replacement of the culture media is required. (ii) The culture media is aspirated using pipetting located at the edge of the microwell array. (iii) Fresh culture is injected using a pipette at the edge of the microwell array. (iv) The supplied fresh culture medium diffuses into the microwell, and nutrients are transferred to the spheroids.

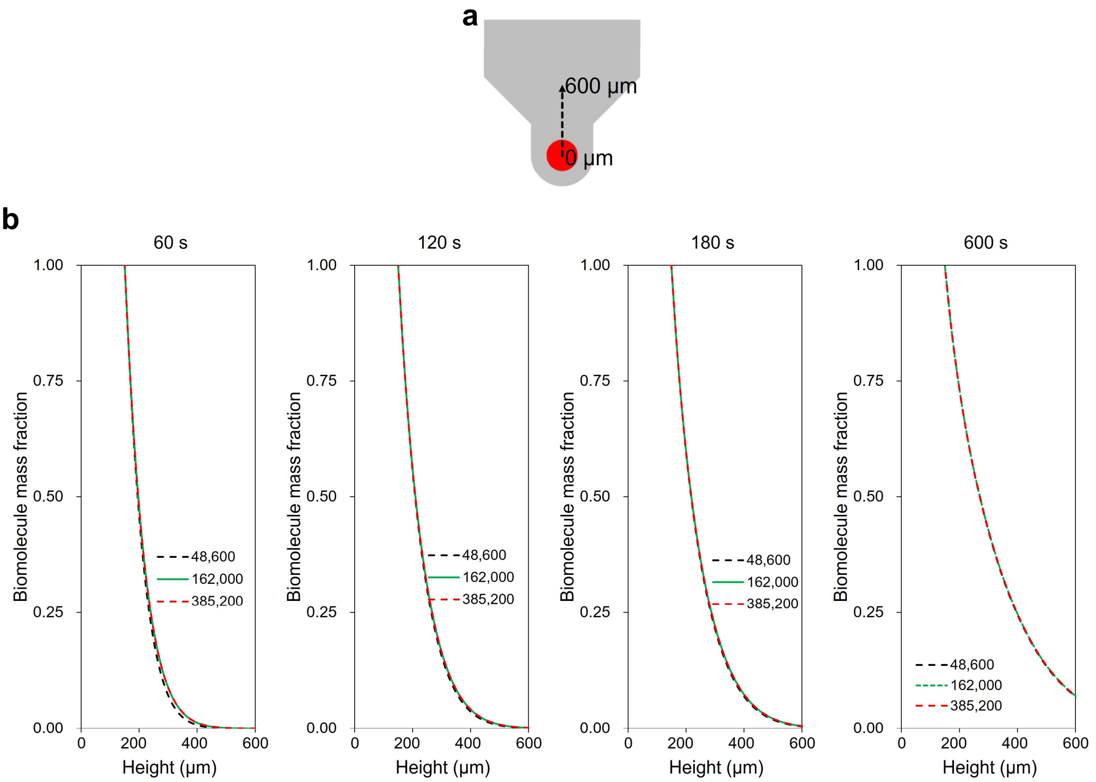

Figure S4. Grid independence test. The biomolecule mass fractions are compared for each grid model depending on time. (a) The mass fraction profiles are predicted using the dashed arrow lines. (b) As time elapses, the mass fraction differences between the grid models gradually reduce; however, the profile of the 48,600-element model is distinguished from those of other models at 60 s.

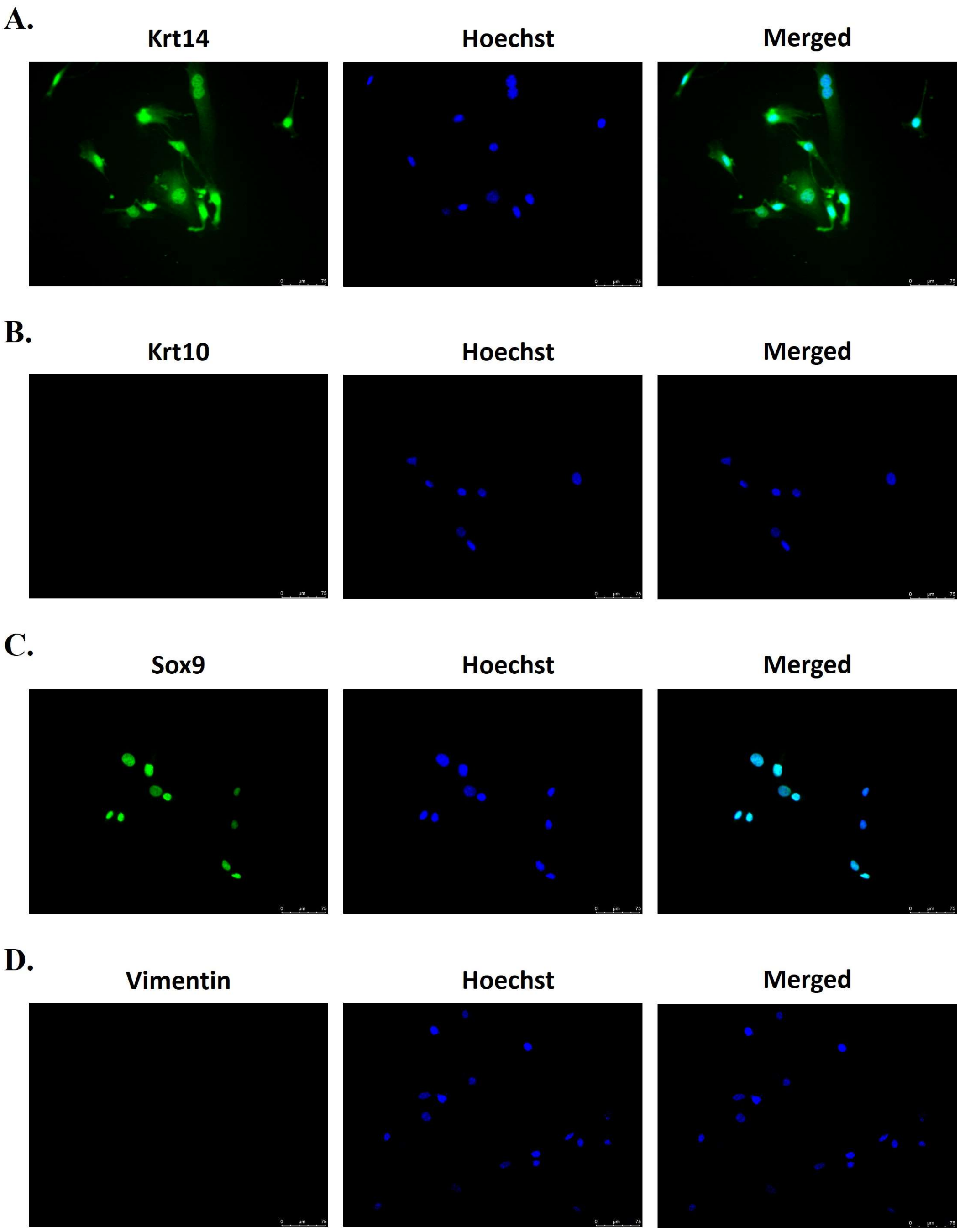

Figure S5. Immunostaining of follicular markers of hair. (A) Protein expression of Krt14 was observed using Krt14 antibody (GTX104124). (B) Krt10, an IFE marker, was not expressed in the isolated HFCs by Krt10 antibody (AB76318). (C) Nucleus expression of Sox9 in HFCs was detected. (D) The expression of Vimentin was absent using Vimentin antibody (SC-6260).

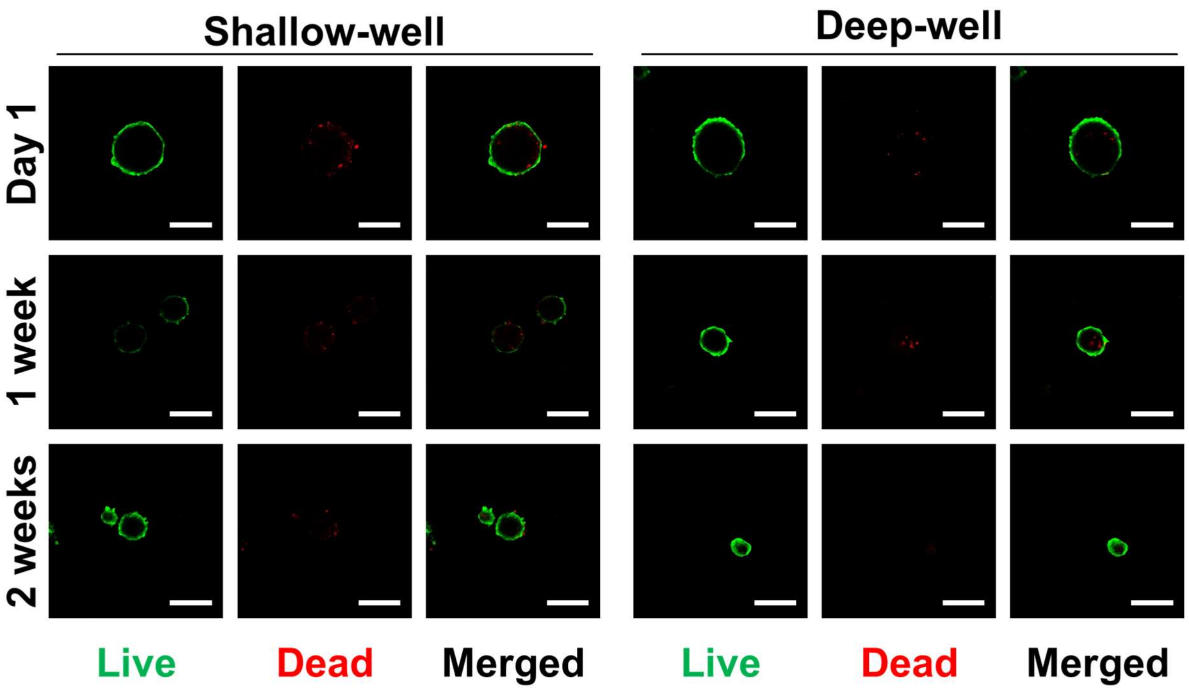

Figure S6. HF spheroids live and dead assay images. It was confirmed that the HF spheroid survived regardless of the depth of the microwell.

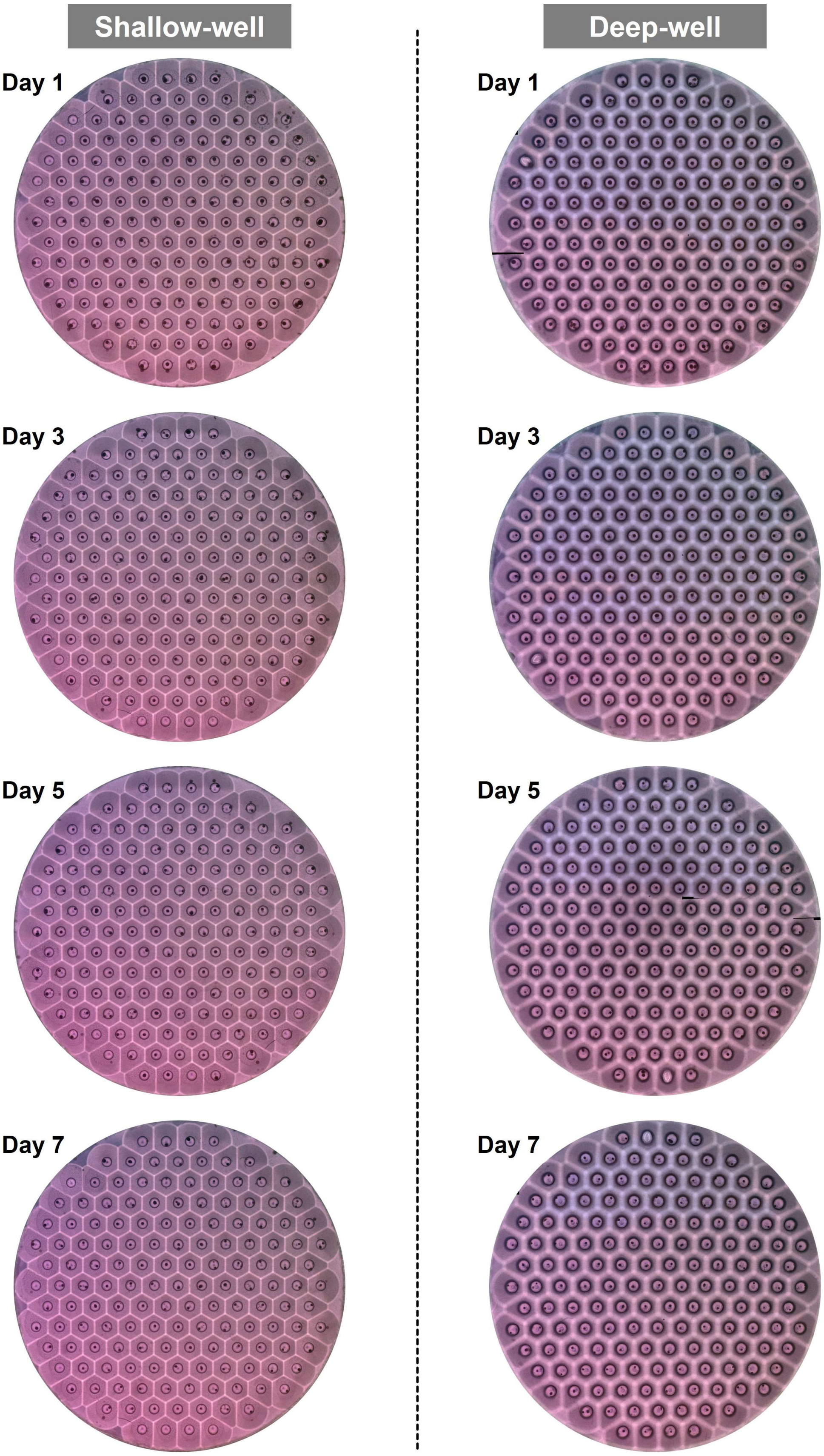

Figure S7. Bright-field images used for the measurement of HF spheroid sizes and positions in the microwells. The spheroids located in the outermost microwells were excluded in the analyses.

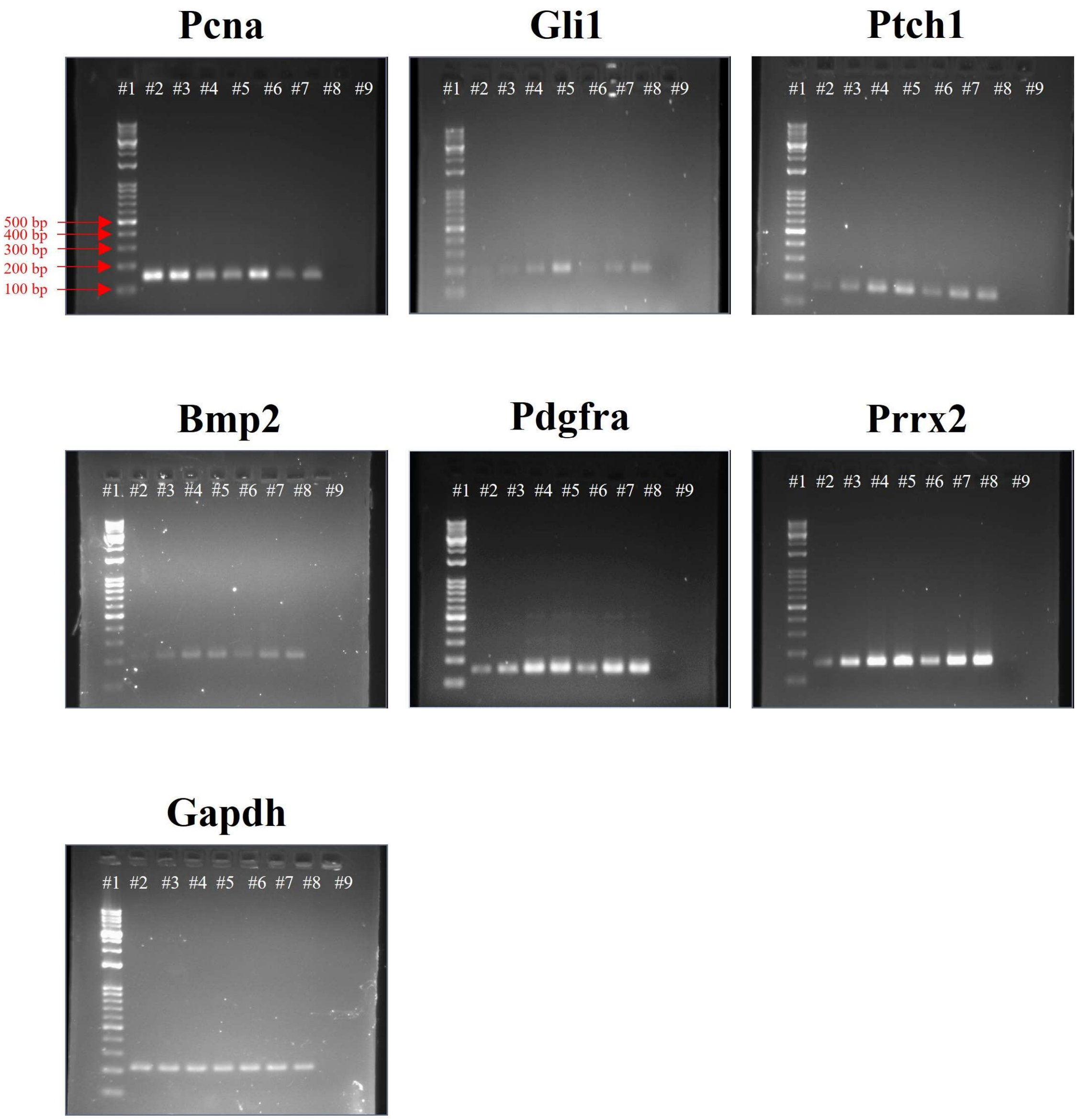

Figure S8. Raw data of agarose gel DNA electrophoresis from figure 7. Lane #1, DNA ladder (DM3200, SMObio); Lane #2, 2D culture sample; Lane #3, day 1 of shallow well; Lane #4, week 1 of shallow well; Lane 5#, week 2 of shallow well; Lane 6#, day 1 of deep well; Lane 7#, week 1 of deep well; Lane 8#, week 2 of deep well; Lane 9#, negative control of PCR.

Table S1. Hair follicle stem cell culture medium composition

Product	Volume (ml)	Working concentration	Catalog number
DMEM	58 ml	Base medium	Gibco 12100-046
DMEM:F12	29 ml		Gibco 11320-033
FBS	10 ml	10%	Gibco 16000-044
B27	2 ml	1X	Gibco 17504-044
P/S	1 ml	1X	Gibco 15140-148
bFGF	varied from stock	20ng/ml	Gibco 100-18B

Table S2. Information of primers in PCR experiments

Target Gene	Primer type	Primer sequence
Bmp2	Forward	5-' CCCAAGACACAGTTCCTTACA -3'
	Reverse	5'- AGCAACACTAGAAGACAGCGG -3'
Gapdh	Forward	5'- GACTCCACGACGTACTCA -3'
	Reverse	5'- GTGGATATTGTTGCCATC -3'
Gli1	Forward	5'- TCAAATTAACAAAGAAGCGGGC -3'
	Reverse	5'- GAATCCTAAAGAAGGGCTCATGG -3'
Pcna	Forward	5'- GGGGTGAAGTTTTCTGCAAGT -3'
	Reverse	5'- TCAGAGCAAACGTTAGGTGAAC -3'
Pdgfra	Forward	5'- CGCGGAACCTCAGAGAGAAT -3'
	Reverse	5'- CCCGGCCCTGTGAGGA -3'
Prrx2	Forward	5'- ACGTGTCCAAGTCTGGTTCC -3'
	Reverse	5'- TCTGGGCTCATCGTGGTAGG -3'
Ptch1	Forward	5'- GCAGATTTCCAAGGGGAAGGC -3'
	Reverse	5'- CACAGCGAAGGCCCCAAATA -3'
